# What Role for Angiogenesis in Childhood Acute Lymphoblastic Leukaemia?

**DOI:** 10.1155/2011/274628

**Published:** 2011-11-09

**Authors:** P. Schneider, I. Dubus, F. Gouel, E. Legrand, J. P. Vannier, M. Vasse

**Affiliations:** ^1^Laboratoire MERCI-EA3829, Rouen University, 22 rue Gambetta, 76000 Rouen, France; ^2^Pediatric Hematology and Oncology, Rouen University Hospital, CHU Charles Nicolle, 1 rue de Germont, 76000 Rouen, France

## Abstract

The role of angiogenesis in acute leukaemia has been discussed since the cloning of the gene of *vascular endothelial growth factor* (VEGF) from the acute myelogenous leukemia cell line (HL60) and, thereafter, when the first studies reported increased bone marrow vascularity and elevation of angiogenic cytokines in acute lymphoblastic leukaemia (ALL). VEGF and *basic fibroblast growth factor* (bFGF) are the major proangiogenic cytokines that have been studied, and evaluation of their prognostic impact in childhood ALL has been reported in several studies, though with controversial results. The antiangiogenic response, contributing to the angiogenic balance, has scarcely been reported. The origin of the factors, their prognostic value, and their relevance as good markers of what really happens in the bone marrow are discussed in this paper. The place of antiangiogenic drugs in ALL has to be defined in the global treatment strategy.

## 1. Angiogenesis in Hematological Malignancies

Angiogenesis is a highly regulated process balanced by inhibitors and stimulators of endothelial cell proliferation, endothelial cell migration, and capillary formation molecules. Cancer cells begin to promote angiogenesis early in tumorigenesis. This angiogenic switch is promoted by oncogene-driven tumor expression of proangiogenic proteins. The key mediator of angiogenesis is a proangiogenic factor, the *vascular endothelial growth factor* (VEGF). VEGF is a homodimeric glycoprotein which stimulates angiogenesis and vascular permeability by interacting with the tyrosine kinase receptor 2 (VEGFR-2 or KDR/Flk-1) and 1 (VEGFR-1 or Flt-1) [1]. These receptors belong to the superfamily of receptor tyrosine kinases (RTKs) and share a common structure with a single transmembrane domain. The central role of the VEGF-R2 in angiogenesis in general and in the development of solid tumors in particular has been widely demonstrated, and it is now considered as the main mediator of VEGFA effect on endothelial cells. Its interaction with VEGF is enhanced by neuropilin-1, which, thereby, has been proposed as a potential target to inhibit VEGF-driven angiogenesis [2].

One other major factor involved is the *basic fibroblast growth factor* (bFGF), a member of the FGF family of proteins, which interacts with tyrosine kinase receptors (FGFR-1 or FGFR-2) to exert mitogenic effects on endothelial, ectoderm-, and mesoderm-derived cells. Beyond its mitogenic activity, bFGF acts synergistically with VEGF in the angiogenic switch [3]. VEGF-A and bFGF are both major stimulators of angiogenesis that are commonly found in malignant tumors. Many other factors are involved in this physiological process and can balance the effects of these major activators of angiogenesis, such as the angiopoietin family [4], endothelins, or adipokines, which are all expressed in the bone marrow (for a review, see [5]). 

It is now well established that solid tumors progress in concert with an induction of tumor angiogenesis which is fundamental for tumor growth and spread. In adults, the physiological angiogenesis is limited to a few specific processes such as wound healing, tissue repair, and the female reproductive cycle [6]. The work of Folkman led to the recognition that angiogenesis plays a major role in tumor development, progression, and metastasis [7]. There is emerging evidence that angiogenesis plays a role in the physiopathology of hematological malignancies as well [8, 9].

Leukemias have been associated with angiogenesis since the acute myeloid leukaemia (AML) cell line HL60 was first used to clone the VEGF gene. High microvessel density in acute leukemias, presence of receptors to proangiogenic factors on leukemic cell lines, and some evidence of prognostic impact of plasmatic levels of VEGF in AML [10] are convincing arguments of the impact of angiogenesis in leukemogenesis.

Beside the direct effect of angiogenic factors on endothelial cells, autocrine and paracrine VEGF/VEGF-related loops were described in hematological malignancies such as acute and chronic leukaemia, myelodysplastic syndromes (MDS), myeloproliferative neoplasms, lymphomas, and multiple myeloma [11]. Recently, neuropilin-1 expression was found increased in the bone marrow of AML and ALL patients when compared to control specimens [12]. It has been described in several medullar cell types as well as in leukemic cell lines [12] and thus may modulate the VEGF autocrine loop that enhances survival of leukemic cells [13]. Though, it remains difficult to establish a clear relation between intra-medullary vascularisation and the expression of angiogenic factors.

## 2. Angiogenesis in Childhood Acute Lymphoblastic Leukaemia

Increased bone marrow vascularity has been reported in chronic and acute leukaemia of myeloid and lymphoid lineages in adults [14–16]. In adult ALL as well, some studies show the role of angiogenesis [14, 17]. In childhood ALL, the published studies are scarce [18, 19].

The first study demonstrating that childhood ALL progression might be accompanied by an increase of bone marrow vascularisation was published by Perez-Atayde et al. [20]. They demonstrated that the bone marrow of these children had increased blood vessel content compared to normal counterparts, suggesting that leukemia might be angiogenesis dependent and raising the possibility for a role of antiangiogenic therapy in the treatment of leukemia. Detailed analysis of bone marrow sections from ALL patients led to the development of a model to illustrate their irregular, albeit abundant, bone marrow vasculature. After this pilot study, the degree of angiogenesis was quantified either by measuring microvessel density (MVD) in bone marrow biopsies or by iron oxide-enhanced magnetic resonance imaging and was found to be increased in both acute and chronic leukemias [18, 21, 22].

## 3. Which Relevant Markers for Intramedullary Angiogenesis Evaluation?

Concerning intramedullary angiogenesis, two types of investigations are performed, either on BM biopsies or in fluid samples, that is, urines plasma, or serum. Among the huge variety of angiogenic markers already identified, discrepancies between the conclusions of published studies were evidenced.

Beside the evaluation of MVD, the work of Perez-Atayde et al. also established that high levels of urinary bFGF were found in all children with newly diagnosed ALL, compared to nonleukemic controls [20]. Several studies have, since then, investigated the protein levels of VEGF and bFGF on different fluids [19, 26, 30]. Others have analysed VEGF-A mRNA levels in bone marrow samples both at diagnosis and at relapse using reverse transcription polymerase chain reaction (RT-PCR) techniques [24, 27]. In all these reports the number of patients was low, and controversial results were found (Table 1). In contrast to the study of Perez-Atayde et al., we observed elevated levels of urinary bFGF only in 54% of patients.

These discrepancies between the different studies could be related to the heterogeneity of the samples analysed: some were performed on serum which contains higher amounts of angiogenic factors than plasma, because of the storage of these molecules in platelets, such as VEGF or endostatin [23, 32]. In addition, circadian variations of angiogenic factors, especially endostatin, were described and have to be considered [33].

Beside its physiological relevance in the angiogenic process, the noninvasive method of detecting urinary levels of this molecule by an ELISA method on urinary samples made it easy to study on patients, especially in children. In the same way, studies in plasma or serum of angiogenic factors are made on samples collected for usual clinical practice and do not require additional samples. In the countries where no bone marrow biopsies are performed at diagnosis of childhood acute leukaemia, diagnosis is made on bone marrow aspirates. Therefore, it does not seem ethical to perform these biopsies which are invasive. But an important question arises concerning the reliability of measures of angiogenic or antiangiogenic factors: is this indirect way of measuring bone marrow angiogenesis a good reflect of the physiology of angiogenesis in leukemogenesis? 

A part of the answer can be given by the study published in 2006 by Veiga et al. who reported their results on BM plasma from ALL patients. They screened BM plasma for VEGF and bFGF by a competitive ELISA method. Similarly to our results on urine samples, they observed that the BM plasma contained elevated levels of bFGF in 58% of ALL patients compared to the BM plasma of normal donors (*P* < 0.05). In contrast, the mean levels of VEGF did not significantly differ between leukemic and normal BM plasma (*P* > 0.1) [34]. These results show that, in a majority of cases, the leukemic BM plasma contained increased levels of bFGF, extending the observations of high bFGF levels in urine or plasma [14, 26]

We previously reported about higher median levels of urinary bFGF in patients than in healthy controls [25]. Interestingly bFGF levels were lower for patients with a bad outcome (relapse or death). This work was extended to a larger number of patients and included measures of urinary VEGF levels which were not higher in patients than controls, but high levels of VEGF indicated poor prognosis with a high relapse rate [26]. 

The angiogenic response in children with ALL is actually demonstrated. The limits of the various studies remain that they hardly reflect the complexity of the *in vivo* balance of pro- and antiangiogenic cytokines interacting as a whole process. If the role of proangiogenic factors is documented, less is known about the antiangiogenic response. Endostatin is a fragment of collagen XVIII having anti angiogenic effect through antimigratory and anti-proliferative properties [35]. In a previous work we focused our attention on endostatin in childhood ALL and observed that the median serum level of endostatin was significantly higher in patients than control at diagnosis as well as in remission. The highest levels were observed in patients with a hyperdiploid karyotype. It is interesting to note that collagen XVIII is mapped to chromosome 21. Thereby, the possible duplication of chromosome 21 in patients with a hyperdiploid karyotype could explain our results [29]. In contrast to urinary levels of bFGF and VEGF, no prognostic value of plasma endostatin can yet be considered. To our knowledge, no other study concerning antiangiogenic factors and childhood ALL has been published since.

## 4. Prognostic Value of These Factors

The first results concerning the prognostic value of angiogenic factors were reported in AML adult patients, showing that high levels of cellular VEGF were significantly correlated with shorter survival in patients with high WBC at diagnosis (*P* < 0.04), whereas no association was found with the plasma levels of bFGF [10]. In B-cell chronic lymphocytic leukaemia, serum levels of VEGF correlated positively with biological markers of prognostic relevance such as ZAP-70 expression, CD38 expression and mutational status of IgV_H_. In this study high circulating VEGF levels reflected an aggressive biological profile [36].

Controversial results were found concerning the association of VEGF or bFGF with usual prognostic factors of ALL and the outcome of patients (Table 1). One of the pediatric studies was performed on 31 children with newly diagnosed ALL and on 22 patients at relapse. Significantly higher VEGF levels were found in patients at relapse, and lower VEGF levels at diagnosis were associated with a longer overall survival [24]. In another study, serum levels of VEGF and bFGF were compared in a group of 31 children with ALL at diagnosis and then in complete remission (CR). The authors reported higher VEGF levels in CR than at diagnosis (26 children/31), suggesting that VEGF could increase when normal hematopoiesis is restored [19].

In a series of children with ALL, we found a correlation between usual poor prognostic features and the interactions between leukemic cells and BM environment. Twenty-three B-lineage ALLs at diagnosis were studied for the urinary secretion of bFGF and VEGF. The levels of bFGF were markedly elevated in ALL children versus controls (median 460 versus 30 pg/mmol creatinine, respectively, *P* < 0.01) but lower for patients with poor prognostic features. In this population there was a significant relation between normal levels of bFGF and high-risk factors (*P* = 0.027). In the same population, median value of VEGF was higher for patients than controls, but without statistic difference. We cultured leukemic cells with and without fibroblasts and observed a reduction of apoptosis rate (annexine V test), especially in patients with high urinary bFGF levels. Among the patients with a bFGF level within the normal range, most show no influence of fibroblasts on apoptosis, suggesting that a subset of highly proliferative leukemias could have a growth independent of the medullary stroma [25].

The role and influence of medullary stroma has been stressed out in a recent study by Norén-Nyström et al. where MVD was analysed at diagnosis in large series of 185 patients. Correlations were found between higher MVD and some specific biological features: T-ALL phenotype, high-hyperdiploid leukemia, and high WBC count in B-ALL. They also showed that B-ALL patients had a worse outcome when having high MVD and high marrow reticulin fibre density [37].

## 5. Origin of the Angiogenic Factors

The angiogenic cytokines can be produced by leukemic and/or bone marrow (BM) microenvironmental cells. 

In a previous work we focused on the origin of pro- and antiangiogenic factors (bFGF, VEGF, endostatin) in patients, trying to identify whether they originate from lymphoblasts or not. Lymphoblast mRNA expression (RT-PCR) has shown that VEGF and endostatin partially originate from lymphoblasts, whereas bFGF seems to have a stromal origin. Quantification in the supernatant of lymphoblasts confirmed these findings in half of the cases studied (Table 2). In a recent work using protein microarrays, we observed that the supernatants of purified lymphoblasts contain only a limited number of angiogenic factors. In a series of patients we found a significant expression, of *insulin growth-factor binding protein 2 *(IGFBP-2), *interleukin-8* (IL8) but also of the *matrix metalloprotease-9* (MMP-9) and its inhibitor* tissue inhibitor of MMP -1* (TIMP-1) (unpublished data).

Therefore, the interaction between leukemic cells and microenvironment is certainly determinant in the production of angiogenic factors. Veiga et al. have shown a contact-dependent interaction between ALL cells of patients and BM endothelium in presence of angiogenic cytokines. This interaction leads to the survival of leukemic cells by maintaining the expression of antiapoptotic genes [38]. 

**Figure 1:**
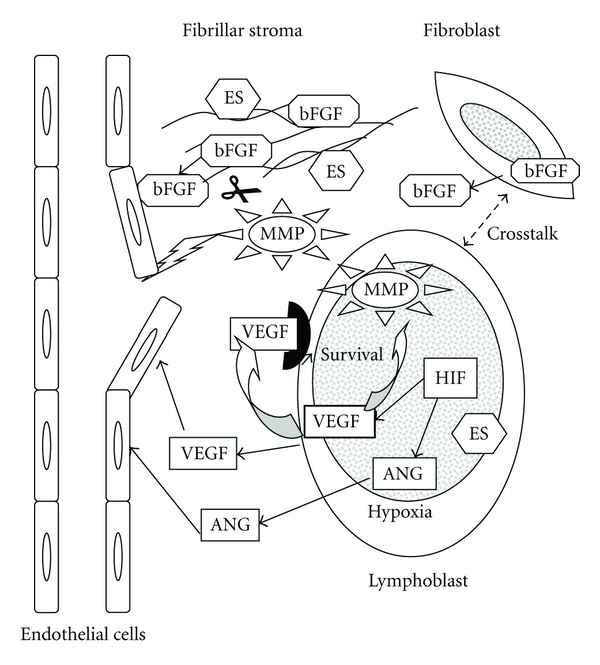
Interactions between leukemic cells (lymphoblasts) and some of the BM components. Leukemic cells and endothelial cells produce *vascular endothelial growth factor* (VEGF), and *basic fibroblast growth factor* (bFGF) is produced by fibroblasts. VEGF plays a key role in vessel growth but also in blood vessel survival. Through its autocrine loop, VEGF enhances lymphoblast survival. The matrix metalloproteases secreted by lymphoblasts degrade the extracellular matrix and liberate growth factors such as bFGF. Hypoxia induces, via the HIF-1*α* subunit, the expression not only of VEGF but also of angiopoietin 2 (ANG). Endostatin (ES) is expressed by leukemic and stromal cells.

The BM microenvironment contains fibroblasts, adipocytes, osteoblasts, in addition to endothelial and hematopoietic cells, which all may interact with leukemic cells and modulate ALL progression. Matrix metalloproteases (MMPs) are a family of zinc-dependent neutral endopeptidases involved in physiological proteolytic degradation of various components of the extracellular matrix which is an important source of pro- and antiangiogenic factors [39]. They participate to the crosstalks between leukemic and stromal cells (Figure 1). We observed that a high secretion of MMP-9 is associated with a poor prognosis in childhood ALL, but there was no direct correlation between the secretion of MMP-9 and urinary bFGF or VEGF [40].

In a study by Suminoe et al., ratios of MMP-mRNAs and TIMPs-mRNAs have been established, and there is a positive correlation between these ratios and the invasivity of leukemic cells, showing the possible role of MMPs in ALL progression [41].

## 6. To a Better Knowledge of Leukemia Angiogenesis: Influence of Hypoxia

In solid tumors, it is known that local hypoxia induces transcriptional regulators, the most prominent of which appears to be *hypoxia-induced factor 1* (HIF-1). It has been demonstrated that hypoxia (about 6% oxygen partial pressure) exists in normal marrows [42] as well as in marrow from patients suffering from acute myeloid leukemia [43]. In addition, the HIF-1*α* subunit is overexpressed in leukemic cell clusters within the bone marrow of children suffering from ALL [27]. Thus, studies on the role of ALL cells in the induction or maintenance of bone marrow angiogenesis may lack physiologic relevance when performed in standard culture conditions (under 21% O_2_). 

For this reason, we developed an *in vitro* model to study of the role of reduced oxygen conditions on the interactions between ALL cells and bone marrow endothelial cells. We used the human pre-B-lymphoblastic Nalm-6 cell line. Cells were cultured either in a conventional incubator, for example, under 21% oxygen pressure and 5% carbon dioxide (normoxic condition), or in a hypoxia chamber with low-oxygen air that contained 5% oxygen and 5% carbon dioxide (hypoxic condition). 

The pattern of angiogenic factors secreted by Nalm-6 cells in normoxic condition was very similar to that observed in cells from patient bone marrow, except that Nalm6 cells do not secrete detectable levels of interleukin-8. Comparison of supernatants from Nalm6 cells in normoxic and hypoxic condition allowed us to identify 3 groups of cytokines: (1) proteins whose secretion was not modified by hypoxia, (2) proteins whose secretion was enhanced by hypoxia, and (3) proteins that were not detectable in supernatants from normoxic condition and were detected in those from hypoxic condition. This model is certainly closer to physiopathological conditions and could help to have a better understanding of the role of each angiogenic factor.

## 7. Place of Antiangiogenic Therapies in the Global Strategy of ALL Treatment

Chemotherapy treatment intensity in childhood ALL is reaching its upper limits regarding induced toxicity versus benefit for the patient, in particular for high-risk patients. Over the past decades, outcome of these patients has widely improved, especially in standard risk groups. Though, high-risk patients and some refractory patients to regular treatment still need new therapeutic approaches. Antiangiogenic drugs are already used in clinical practice for the treatment of metastatic breast cancer and advanced or metastatic renal tumors and show interesting results in association with conventional therapy [44]. In haematological malignancies, the use of thalidomide in multiple myeloma is one of the pioneers in antiangiogenic treatments [45]. Some antiangiogenic drugs have been suggested as a supplementary treatment alternative in acute leukaemia, and new drugs are presently under investigation in clinical trials in refractory AML (NCI clinical trials). But the place of these treatments still has to be defined. In a feasibility study, bevacizumab (Avastin, Genetech) was administered to adult patients with refractory AML and resulted in modest clinical benefit [46]. 

In our work concerning childhood ALL, we found an association between normal bFGF levels and poor outcome suggesting that some proliferative forms of leukaemia may proliferate without the influence of stromal microenvironnement [25]. On the other hand, VEGF levels are significantly increased for relapsing patients, showing that angiogenic factors are differently involved in the evolution of the disease [26]. In a risk-adapted strategy, it is still necessary to define properly which group of children will benefit from these new agents. As an example, Norén-Nyström et al. have identified some biological subgroups of childhood ALL patients, like T-ALL which are usually more aggressive forms of ALL, with high MVD scores [37]. They suggest that these subgroups should probably be the first to be considered for antiangiogenic therapies.

Beside direct effect of antiangiogenic drugs, the autocrine loop of VEGF enhances survival of leukemic cells and might be an interesting target for anti-VEGF molecules, that is, in aggressive forms of childhood ALL. Moreover, one must point out that targeting VEGF-A sometimes results in adaptative resistance as it has been observed in solid tumors [47]. Drug development targeting other angiogenic factors such as the angiopoietin-Tie2 pathway [48] or preventing the interaction between leukemic cells and endothelium [49] may offer new therapeutic combinations to improve clinical responses.

## 8. Conclusions

Targeting VEGF is perhaps an interesting treatment strategy, not only for the inhibition of angiogenesis but to disrupt the autocrine loop associated with the survival and invasivity of leukemic cells. Indeed it has been shown that VEGF induce the secretion of MMP-2 and MMP-9 which contribute to the lymphoblasts invasivity [13]. We also demonstrated that secretion of MMP-9 is a poor prognostic factor in childhood ALL [40]. Therefore, inhibition of VEGF could be of potential interest to limit aggressivity of proliferative forms of childhood ALL. In agreement with this hypothesis, it was shown that some VEGF polymorphisms were associated with high risk of relapse in ALL children [50]. However, it was not precised in this work if these polymorphisms were associated with different levels of MVD or independent of angiogenesis.

At least at this time our knowledge of angiogenic process in childhood ALL remains poorly understood. It remains unclear which parameter is the most relevant as angiogenic marker and which sample (BM, urine, plasma, or serum) should be analysed. Most of the studies focused on bFGF and VEGF, but many other angiogenic factors can modulate bone marrow vascularisation. In other haematological malignancies, VEGF receptors have been widely explored, and the role of angiopoietin-2 has been clearly identified in AML [51]. At this time, the analysis of factors one by one is not a satisfying approach, and global strategies are needed, such as proteomic and genomic methodologies.

Considering the low incidence of childhood ALL, multicentric studies are mandatory to analyse the influence of angiogenesis in leukemogenesis and the potential interest of angiogenic therapies.

## Figures and Tables

**Table 1:** Main studies concerning the prognostic value of bFGF and VEGF in adult (A) and childhood (C) ALL.

Authors, journal, and publication year	*N*. patients	Type of samples	Measured criteria	Prognostic value
Aguayo et al. *Blood*, 2000 [14]	A : 28	BM [20] and Plasma [23] (diagnosis)	MVD and plasmatic levels of VEGF and bFGF	Not determined. ↗ MVD; no high levels of VEGF in LAL

Koomagi et al., *Clin Cancer Res*, 2001 [24]	C : 53	BM (RT-PCR), diagnosis [25], relapse [22]	VEGF levels, outcome	↘ VEGF correlated to better overall survival

Yetgin et al., *Leukemia Lymphoma*, 2001 [19]	C : 31	Serum (VEGF & bFGF) (diagnosis and CR)	Correlations with prognosis criteria (HMG, WBC, Hb, platelets, phenotype)	Positive correlation, only with platelets

Pulè et al., *Br J Hematol*, 2002 [18]	C : 41	BM (diagnosis and CR)	MVD & correlations with outcome (relapse) and prognosis criteria (age, sex, cytogenetics, phenotype)	No correlation

Schneider et al., *Br J Hematol,* 2003 [26]	C : 39	Urine (diagnosis)	Levels of urinary bFGF and VEGF levels & outcome	↘ bFGF and/or ↗ VEGF significantly associated with poor outcome

Wellmann et al., *Leukemia*, 2004 [27]	C : 18 C : 6	BM (diagnosis) Plasma (relapse)	Expression of VEGF & VEGF-R & HIF-1*α* by immunostaining; RT-PCR on BM-extracted RNA	↗VEGF and HIF-1*α* shorter DFS ↗VEGF if treatment resistance (MRD+)

Faderl et al. *Blood*, 2005 [17]	A : 95	Plasma (diagnosis)	Relative risk of death (RR) according to VEGF levels	RR of death ×8 if VEGF <19.1 pg/mL

Avramis et al., *Clinic Cancer Res*, 2006 [28]	C : 17	Serum (diagnosis, d14, d28)	Evolution of VEGF levels during induction; correlation with EFS	Positive predictive value of relapse if ↗ VEGF

Schneider et al.*, Leuk Res, *2007 [29]	C : 33	Urine and plasma (diagnosis, relapse)	Levels of endostatin, bFGF, VEGF	↗ plasma VEGF at relapse No prognostic value of endostatin levels

Lyu et al., *Yonsei Med J*, 2007 [30]	C : 33	BM Plasma (diagnosis and relapse)	Levels of VEGF & bFGF, correlations with WBC and age	Higher bFGF and VEGF levels in relapse. No correlations

Stachel et al., *Oncology Reports*, 2007 [31]	C : 46	BM (diagnosis)	RT-PCR on BM-extracted RNA; correlations with relapse and survival	↗ VEGF correlated to late relapse

BM: bone marrow; MVD: microvessel density; C: children; A: adults; D: at diagnosis; CR: complete remission; RR: relative risk; EFS: event-free survival; bFGF: basic fibroblast growth factor; VEGF: vascular endothelial growth factor; HIF-1*α*: subunit 1*α* of hypoxia-induced factor 1; MRD: minimal residual disease.

**Table 2:** Expression of endostatin, *vascular endothelial growth factor* (VEGF), and *basic fibroblast growth factor* (bFGF) by lymphoblasts (RT-PCR) in a group of 24 patients. The protein was measured by ELISA method in 28 patients and was expressed in half of the cases studied, for endostatin and VEGF. We found no expression of bFGF by the lymphoblasts, neither bFGF mRNA nor protein expression [29].

	mRNA (RT-PCR)	Protein (ELISA)
Endostatin	19/24	14/28
VEGF	24/24	6/12
bFGF	0	0
